# Mandibular reconstruction after excision of recurrent odontogenic keratocyst using a novel mandibular distraction osteogenesis method– a case report

**DOI:** 10.1186/s13005-023-00367-0

**Published:** 2023-06-02

**Authors:** Haiyun Lin, Xiaoxia Zhong, Nuo Zhou, Xuan-Ping Huang

**Affiliations:** 1 Department of Oral and Maxillofacial Surgery, College & Hospital of Stomatology, Guangxi Medical University, Nanning, 530021 People’s Republic of China; 2 Medical Scientific Research Center, College of Stomatology, Guangxi Medical University, Nanning, 530021 People’s Republic of China; 3 Guangxi Key Laboratory of Oral and Maxillofacial Rehabilitation and Reconstruction; Guangxi Key Laboratory of Oral and Maxillofacial Surgery Disease Treatment; Guangxi Clinical Research Center for Craniofacial Deformity, Guangxi Health Commission Key laboratory of prevention and treatment for oral infectious diseases, College of Stomatology, Guangxi Medical University, Nanning, 530021 People’s Republic of China; 4 Department of Prosthodontics, College & Hospital of Stomatology, Guangxi Medical University, Nanning, 530021 People’s Republic of China; 5 Orthognathic Surgery Center, Affiliated Stomatological Hospital of Guangxi Medical University, No. 10, Shuangyong Road, Qingxiu District, Nanning, Guangxi Zhuang Autonomous Region People’s Republic of China

**Keywords:** Odontogenic keratocyst, Mandibular segmental defect, Reconstruction, Distraction osteogenesis, Case report

## Abstract

**Background:**

Odontogenic keratocyst is one of the most common benign odontogenic neoplasms with a high recurrence rate. Its resection has the potential to lead to mandibular segmental defects. In this case report, we describe a patient with odontogenic keratocyst who underwent radical resection using a novel distraction osteogenesis (DO) method to reconstruct mandibular segmental defect.

**Case presentation:**

This case report describes a 19-year-old woman with odontogenic keratocyst of the mandible that recurred after multiple curettages and eventually necessitated radical resection. Mandibular segmental defect after radical resection was reconstructed using a novel DO method that involved directly contacting the segment ends of the defect without the transport disk. However, the distractor broke during the retention period, and a molding titanium plate was used for fixation. This novel distraction method achieved mandibular reconstruction and restored mandibular function and contour.

## Background

Odontogenic keratocyst (OKC) is a common cystic lesion occurring in the jaws. It shows aggressive behavior, tendency to recur, rapid growth, and susceptibility to invade surrounding tissues [1]. The recurrence rate of OKC is relatively high after curettage or enucleation and low after jaw resection. However, jaw resection causes jaw defects, necessitating jaw reconstruction to restore contour and function.

Distraction osteogenesis (DO), as an endogenous tissue engineering technique, has been widely used in orthognathic surgery to correct oral and maxillofacial deformities and repair bone defects after tumor ablative surgery, facial trauma, and inflammatory and infection diseases by applying transport disk DO (TDDO) [2–4].

TDDO offers an advantage in bone defect reconstruction of avoiding complications at the second surgical and donor sites [5]. However, complications such as transport disk necrosis, resorption at the boundary of the compressed end of the transport disk, suboptimal osteogenesis between the transport disk and the jaw segment, and movement of the teeth on the transport disk with distraction are inevitable [5, 6]. Compared to monofocal DO, TDDO is a more complicated procedure because osteotomies at a bone segment at one or both ends of the bone defect are required to form transport disks and because the distractor occasionally requires a customized design.

No distraction methods have been reported that involved directly contacting the segment ends of the defect without making a transport disk during small surgical repair of segmental jaw defects created after removal of benign tumors of the mandibular body, such as odontogenic keratocyst (OKC) or ameloblastoma.

Here, we report a case of OKC of the mandible that recurred after multiple curettages and eventually necessitated radical resection. A novel DO method without the transport disk was used for mandibular repair. However, the distractor broke during the retention period, and a molding titanium plate was used for fixation. This novel distraction method could achieve mandibular reconstruction and restoration of function and contour. To the best of our knowledge, no similar cases have been reported before.

## Case presentation

**Fig. 1:**
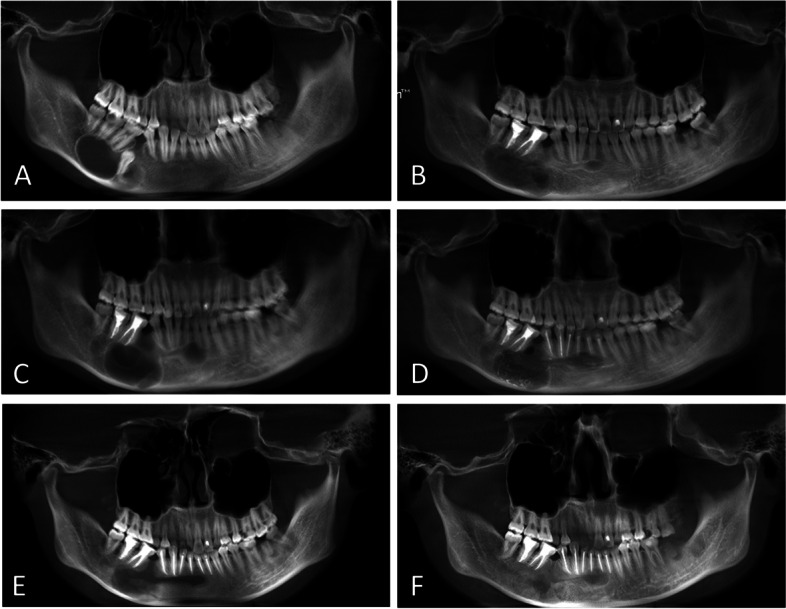
Panoramic radiographs showing recurrence after the first curettage and the mandibular lesion gradually decreasing in size after the second curettage. **A** Before curettage. **B** Seven months after curettage. **C** Twenty-one months after curettage. **D** Immediately after the second curettage. **E** Four months after the second curettage. **F** Twelve months after the second curettage

**Fig. 2:**
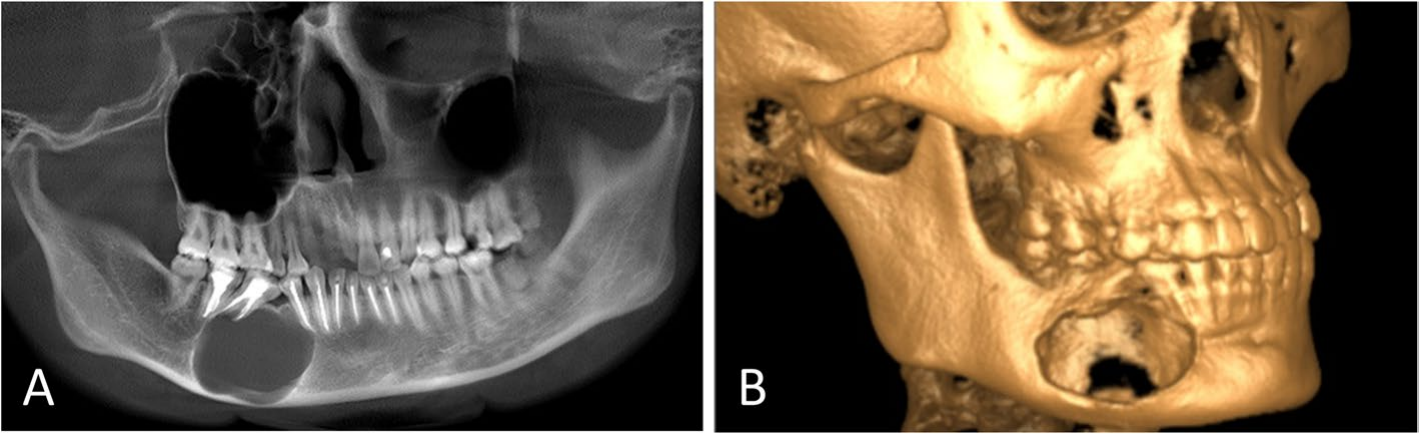
Three years after the second curettage without recurrence at the anterior end of the lesion. **A** Panoramic radiograph showing an enlarged lesion in the right mandibular body. **B** Computed tomography showing buccolingual bone plate defects

A 19-year-old woman was referred to our department because of a cystic lesion in the right mandible incidentally found on a radiograph obtain during oral examination without symptoms. The right face showed no swelling. Oral examination revealed a slight swelling of the mandibular body, missing mandibular right second premolar, inclination of the mandibular right first premolar and first molar, and no numbness in the lower lip. The associated oral mucosa was not swollen. An oval radiolucent lesion was developing along the long axis of the right mandibular body involving the roots of the mandibular right first and second molars with an impacted mandibular right second premolar at the anterior end of the lesion (Fig. 1A). The preliminary radiographic diagnosis was OKC (Fig. 1A). Curettage of the lesion was performed after root canal therapy of the mandibular right first and second molars in January 2016 (Fig. 1B). Histopathologic findings were consistent with OKC. Postoperative reexamination revealed new bone formation at the posterior end of the cyst in the right mandible with a reduced cystic cavity at 7 (Fig. 1C) and 21 months after the curettage (Fig. 1D). However, the anterior end of the lesion extended into the mandibular left lateral incisor and was enlarged. Subsequently, repeat curettage of the lesions was performed. The lesions reduced in size at 4 months (Fig. 1F) and 1 year postoperatively (Fig. 1G). Subsequently, the patient was lost to follow-up for approximately 2 years owing to pregnancy and childbirth but visited again because of right mandibular expansion in August 2020. The cystic lesion in the right mandibular body had enlarged with visible buccolingual bone plate defects (Fig. 2A, B). No recurrence was seen at the anterior end of the lesion. The patient was advised to undergo repeat curettage or complete surgical excision with repair. The patient requested complete surgical excision with planned immediate reconstruction owing to the concern of recurrence after curettage. After fully informing the patient about the extent of bone resection and advantages and disadvantages of autogenous bone grafting and DO, consent was obtained for DO for mandibular reconstruction.

**Fig. 3:**
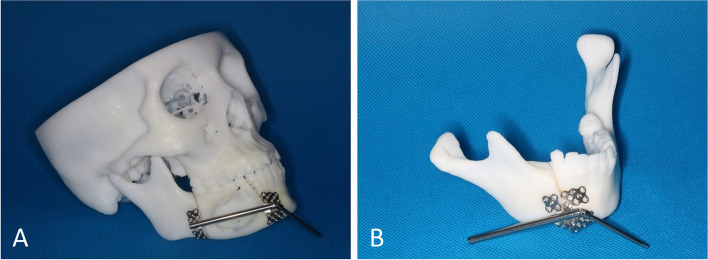
Simulated surgeries on 3D-printed mandible models. **A** Determination of the extent of surgical resection and distance and direction of distraction with the distractor bent preoperatively. **B** Closure of the distractor after resection

**Fig. 4:**
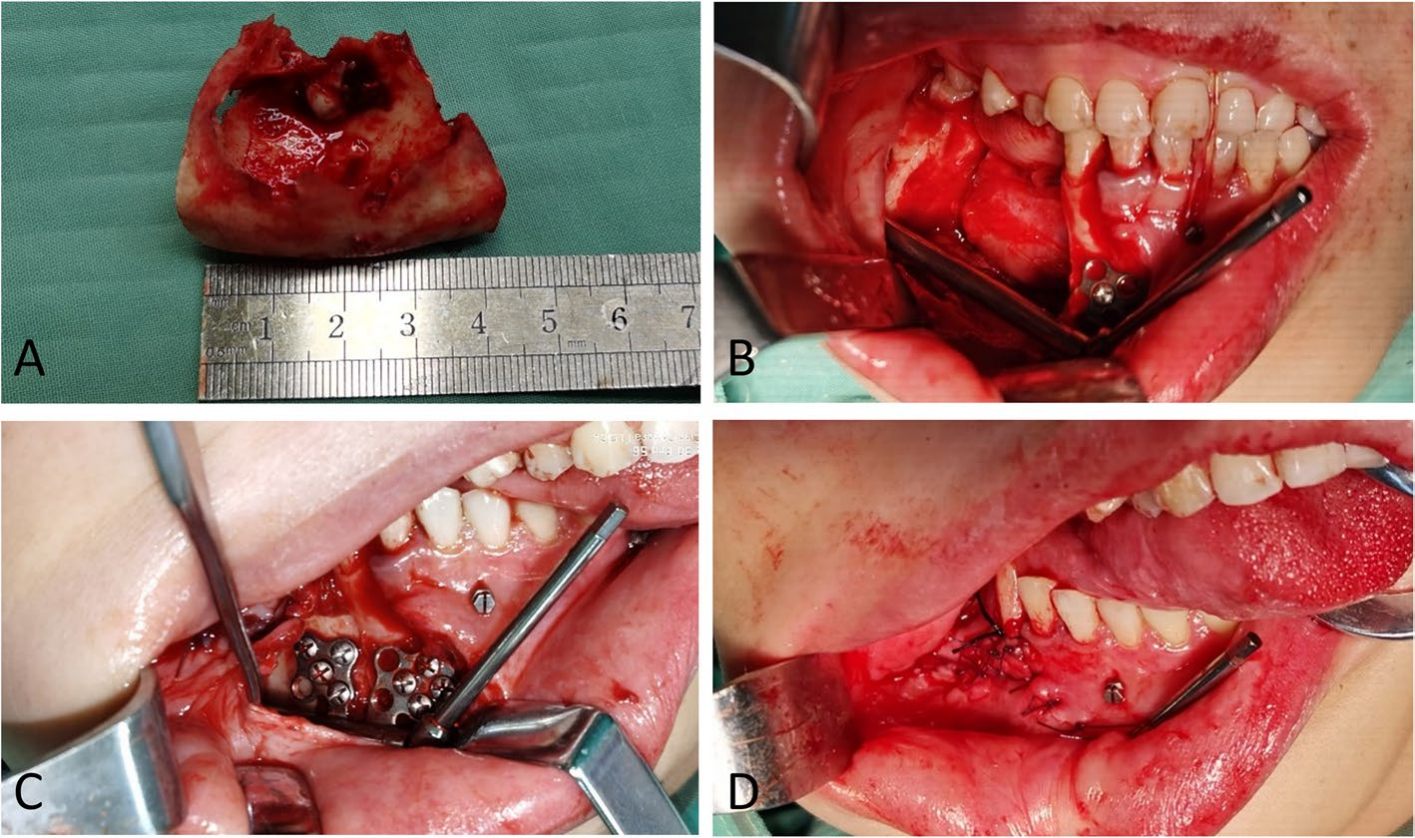
**A** Resected mandibular odontogenic keratocyst. **B** Distractor replaced according to the predetermined position. **C** Closure of the distractor. **D** Malocclusion with mandibular midline deviation and chin retrusion observed after wound suturing

**Fig. 5:**
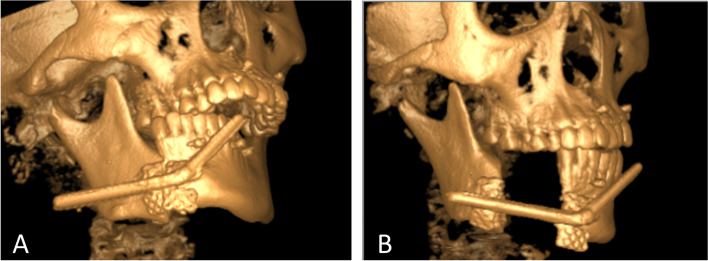
Cone-beam computed tomography showing changes before and after mandibular distraction. **A** Before distraction. **B** Immediately after mandible advancement

**Fig. 6:**
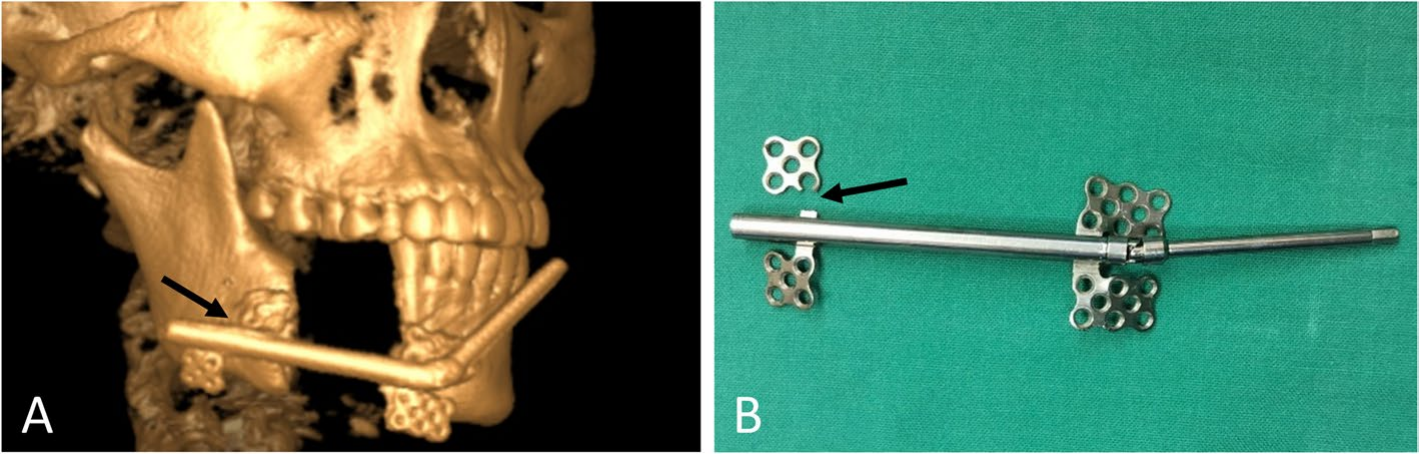
**A** Cone-beam computed tomography showing breakage of the distractor at the joint between the bone anchorage plate and the extension rod at 3 weeks of fixation period. **B** The distractor was removed

**Fig. 7:**
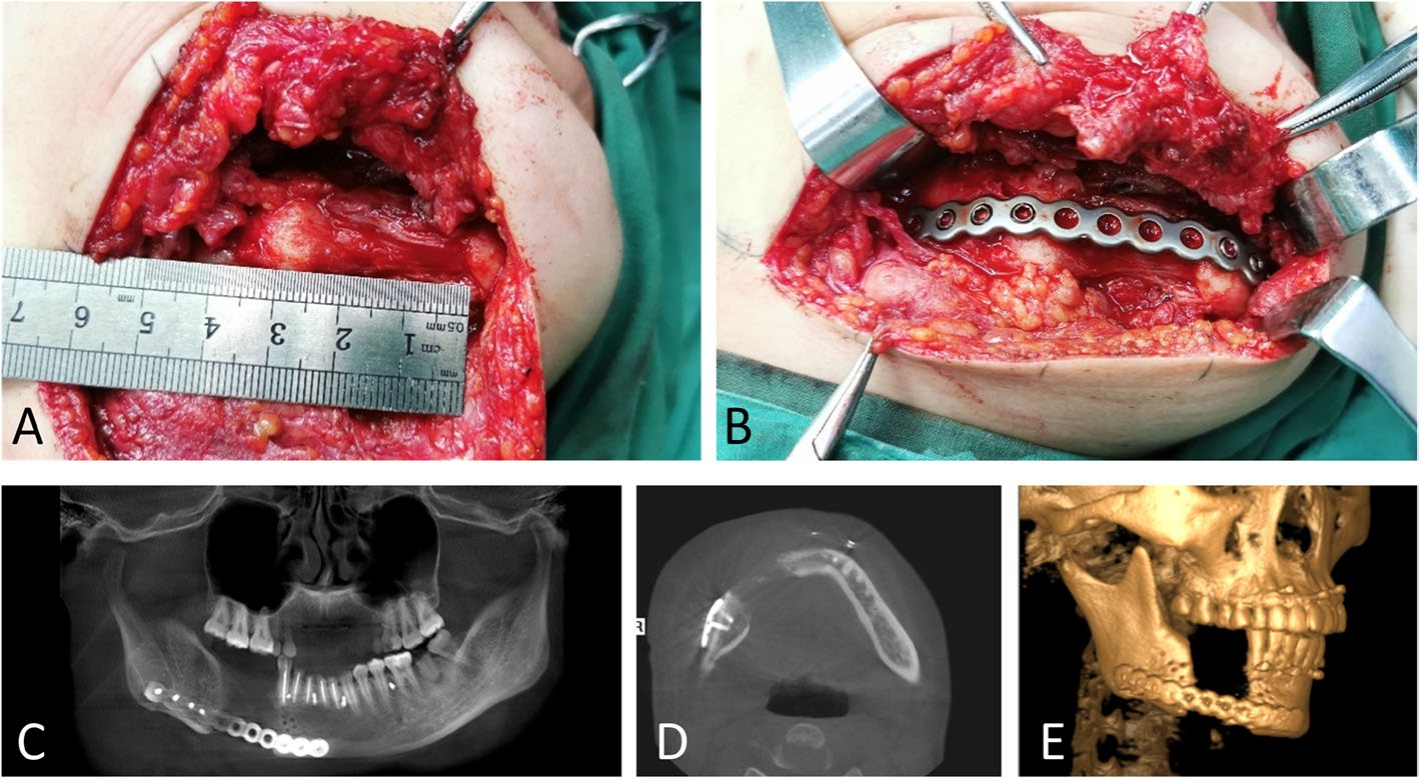
Intraoperative photographs and cone-beam computed tomography showing Incomplete ossification during the distraction area. **A**–**B** Intraoperative photographs. **C**–**E** Cone-beam computed tomography immediately after placing the molding titanium plate

**Fig. 8:**
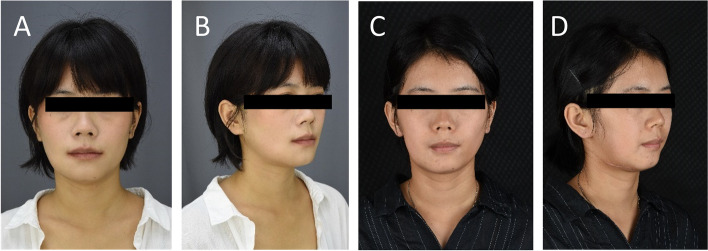
Facial photographs showing no gross facial asymmetry or deformation. **A** & **B** Before mandibular segmental osteotomy. **C** & **D** Seven months after placing the molding titanium plate

**Fig. 9:**
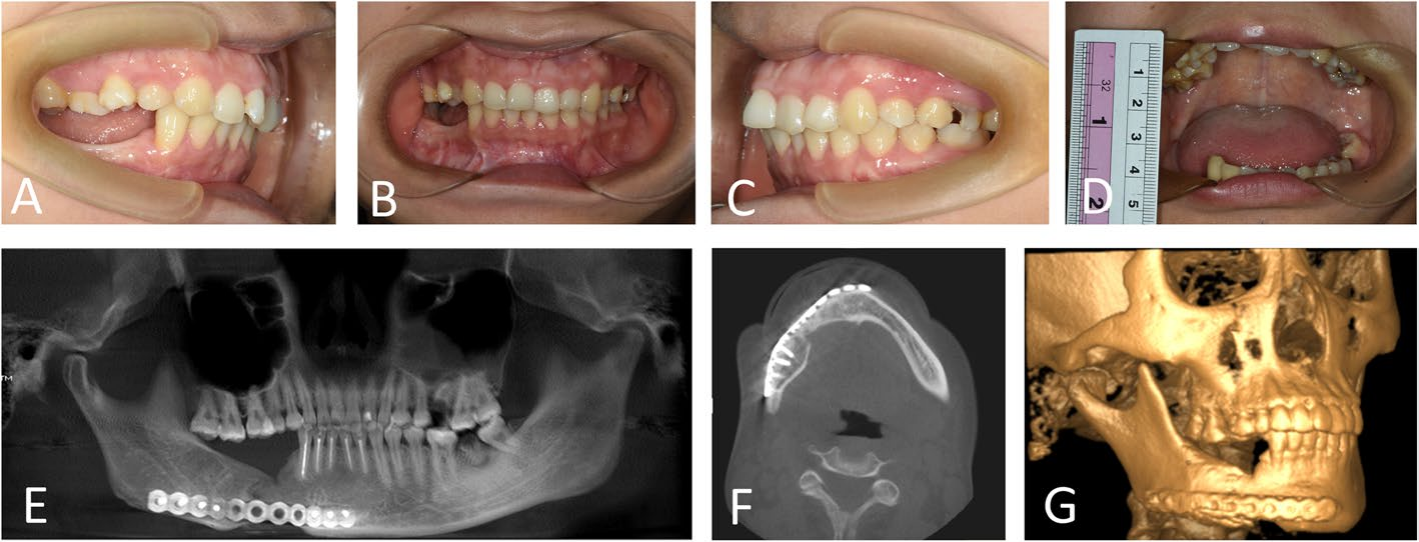
Surgical outcomes showing new bone formation in the distraction area and no abnormalities in mouth opening or occlusal relationship. **A**–**C** Occlusal relationship. **D** Measurement of mouth-opening. **E**–**G** Cone-beam computed tomography 21 months after placing the molding titanium plate

Simulated surgeries were performed on 3D-printed mandible models based on computed tomography data to determine the extent of surgical resection and distance and direction of distraction (Fig. 3A, B). The distractor was bent preoperatively according to the model. Under general anesthesia, a mucoperiosteal flap was raised with the intraoral vestibular sulcus approach to expose the right mandible. An osteotomy line was marked at the boundary of the mass according to the preoperative design, and the pre-bent distractor was fit onto the bone surface with two titanium nails spotted on each of the anterior and posterior arms of the distractor (Fig. 4B). The titanium nails and distractor were then removed, and the lingual periosteum was carefully preserved during removal of the mandible. The distractor was repositioned at the designated position and rotated such that the anterior and posterior segments of the mandible were close (Fig. 4C). The incision was sutured. An endotracheal tube was placed for 3 days to avoid upper airway obstruction possibly caused by retropulsion of the mandible and postoperative swelling. The patient was fed via intravenous nutrition and a gastric tube for 4 days postoperatively, followed by a liquid diet. After a 7-day latent period, the distraction was activated at a rate of 1 mm/day for 32 consecutive days (Fig. 5A, B). At three weeks of fixation period, sudden mandibular midline deviation and tooth malposition presented. Cone-bone computed tomography showed the breakage of the distractor (Fig. 6A). Subsequently, the distractor was exposed and removed via an incision with the right submandibular approach under general anesthesia (Fig. 6B). The distraction area had not completely ossified. The boundary between the distraction area and the mandible was clear, and the width of the distraction area was approximately 25 mm (Fig. 7A). The wound was closed after placing the molding titanium plate (Fig. 7B). Postoperative cone beam computed tomography showed Incomplete ossification during the distraction area (Fig. 7C–E). The patient was followed-up for 21 months after placing the molding titanium plate with no evidence of recurrence. No gross facial asymmetry or deformation was observed (Fig. 8A–D). The patient reported of no complaints regarding eating or articulation. The mouth opening and occlusal relationship showed no abnormalities (Fig. 9A–D). New bone formation could be seen in the distraction area, and the height and width of the reconstructed mandible were acceptable (Fig. 9E–G). The right condyle appeared anteriorly displaced (Fig. 9E, G). Examination of the bilateral temporomandibular joints revealed no clicking or pain. Table 1 depicts the timeline of the present case.

**Table 1:** Timeline of the present case

Date	Appointment	Findings or Procedures
1/2016	First dental examination	Curettage of OKC
8/2016	7-months after curettage	The lesion reduced in size
10/2017	21-months after curettage	Recurrence was found
11/2017		Second curettage procedure
3/2018	4-months after the second curettage	The lesion reduced in size
11/2018	12-months after the second curettage	The lesion reduced in size
8/2020	33-months after the second curettage	Recurrence was found, resection of OKC, replacement of distractor
24/9/2020–26/10/2020	Distraction period	Distraction
11/2020	Breakage of the distractor	Distractor was removed and the molding titanium plate was placed
8/2022	21-months after placement of molding titanium plate	No evidence of recurrence, new bone formation in the distraction area

## Discussion

The characteristic features of OKC include locally aggressive behavior, a high recurrence rate, and propensity for proliferation [1, 7]. The treatment of OKC remains controversial. Curettage is the most commonly used method but is associated with a high recurrence rate [8]. Indications for radical resection of OKC include multiple recurrences, aggressive behavior with perforation of the cortical plates of the jaws or extension into the adjacent soft tissues, and extensive lesions [7, 8]. Radical resection should be limited to OKCs that have recurred more than twice or undergone malignant transformation [9, 10]. Our patient developed recurrences after two curettages, with buccolingual bone plate destruction at the second relapse. Therefore, radical resection was performed after communicating with the patient. No recurrence was found at nearly 2-years postoperative follow-up, and a longer follow-up is warranted.

Mandibular segmental defects caused by radial resection affect esthetics and function, and simultaneous reconstruction is usually performed to restore mandibular continuity. Autogenous bone grafts obtained from the iliac crest, fibula, and ribs have been used for bone defect repair and reconstruction. However, there are risks of graft failure and donor site complications, such as bleeding, inflammation, infection, nerve injury, and chronic pain [11]. DO is a technique that utilizes the potential of the autogenous bone for regeneration. It has advantages in bone defect reconstruction of no requirement of bone grafting, avoiding complications at the second surgical and donor sites, less trauma, and high osteogenesis quality [5]. In the present case,the preoperative design helped to ensure precise contact of the anterior and posterior segments of the mandible after closure of the distractor. This precise contact was similar to an osteotomy suture created during osteotomy in DO. Although a transient mandibular deformity was present before completion of distraction, creation of a transport disk was not required, and the surgical procedure was simpler, avoiding the complication associated with transport DO. The temporomandibular joint is a linkage joint, where small mandibular segmental defects can achieve adequate contact between the anterior and posterior bone segments through joint rotation and sliding, although large mandibular and maxillary defects cannot.

Common mandibular DO complications include infection, soft tissue complications, insufficient vector control, temporary inferior alveolar nerve disturbances, device-related complications, mandibular fractures, insufficient bone formation, and temporomandibular joint-related complications [12]. Temporomandibular joint-related complications include temporary reduction in joint mobility that is often reversible. Ankylosis of the mandibular condyle and joint dislocations are rare complications [13, 14]. In the present case, the mandibular condyle was anteriorly dislocated immediately after placement of the distractor, possibly associated with anterior displacement of the bone mass at the proximal end of the mandible postoperatively affected by the surrounding muscles. The mandibular defect was approximately 42 mm in size after excision of the OKC of the mandible. However, after 32 days of distraction, the occlusal relationship was almost at the preoperative level. Continued distraction might have caused the malocclusion; therefore, the distraction was ended. The distraction gap had retracted after breakage of the distractor. Postoperatively, the condyle had dislocated anteriorly. Mouth opening and the bilateral temporomandibular joints showed no abnormalities 21 months after placing the molding titanium plate, possibly associated with joint re-adaptation at the new position.

Breakage of the distractor is a relatively rare complication [15–18] and associated with poor quality of the distractor, inappropriate distractor vector, wrong distraction protocol, tissue resistance, and abnormal chewing habits [16]. In the present case, the distractor broke during the consolidation period, possibly related to the excessive distraction distance, premature and excessive forces exerted on the mandible, and lack of traction forces. Although intermaxillary traction was provided for approximately 1 week after the consolidation period and liquid diet was advised, these measures might not be sufficient, and increased mouth opening, chewing, and other activities of the patient after removal of intermaxillary traction could lead to excessive stress. Intermaxillary traction should be reinforced after consolidation of distraction to reduce jaw movements and occlusal forces. As the mechanical stress at the distracted gap is concentrated on the joint of the bone anchorage plate and extension rod of the distractor, the joint has a risk of breakage before bone stability by bone regeneration [18]. Therefore, surgeons should pay close attention to this mechanically weak area and avoid unnecessary frequent bending to adapt the bone surface, which may directly weaken the joint [18]. Mechanical resistance is necessary to be considered during the design of the distractor. Strong distraction devices are necessary to reduce the potential risk for device breakage.

Reoperation is usually required to replace a broken distractor during distraction [16, 18]. Aikawa et al. [18] reported a case of distractor breakage observed 3 months after bilateral maxillary distraction without reoperation for fixation, orthodontic treatment was used to effectively preserve the maxillary position. In the present case, the breakage occurred approximately at three weeks of fixation period, when there were lack of new mineralized bone, insufficient hardness in the distraction area, and movements such as chewing, thus moving the bone segments in the distraction gap and resulting in nonunion. Intraoperative findings showed that although the retraction gap had not mineralized, distraction of the anterior and posterior bone blocks was difficult. Therefore, a molding titanium plate was used for refixation to guarantee stability of bone segments. In the present case, a distraction gap of approximately 32 mm was obtained at the end of distraction; however, only 25 mm was measured at the time of the secondary surgery. This might be related to the failure to perform timely surgery after the distractor breakage, displacement of the bone segments, and retraction of tissues before and after the retraction gap during mandibular movements. Nevertheless, the occlusal relationship and midline were maintained in a relatively ideal position with intermaxillary traction postoperatively. After fixation with a molding titanium plate, the distraction gap could gradually become osteogenic.

## Conclusions

Mandibular segmental defect repair was achieved by this novel distraction method that involved directly contacting the segment ends of the defect without making a transport disk during surgery. Complications associated with the transport disk of TDDO were avoided. However, special attention should be paid to the complication of anterior joint displacement. Strong distraction devices and more prolonged restricted mandibular movement are necessary to reduce the potential risk of device breakage.

## Data Availability

Not applicable.

## References

[1] Titinchi F (2021). Novel recurrence risk stratification of odontogenic keratocysts: a systematic review. Oral Dis.

[2] Nanjappa M, Natashekara M, Sendil Kumar C, Kumaraswamy SV, Keerthi R, Ashwin DP, Gopinath AL (2011). “Transport distraction osteogenesis for reconstruction of mandibular defects”: our experience. J Maxillofac Oral Surg.

[3] Neelakandan RS, Bhargava D (2012). Transport distraction osteogenesis for maxillomandibular reconstruction: current concepts and applications. J Maxillofac Oral Surg.

[4] Balaji SM (2016). Total reconstruction of mandible by transport distraction after complete resection for benign and malignant tumors. Indian J Dent Res.

[5] Li T, Man Y, Bi R, Jiang N, Li Y, Zhu S (2017). Reconstruction of mandibular segmental detects using transport disk distraction osteogenesis. J Craniofac Surg.

[6] Castro-Núñez J, González MD (2013). Maxillary reconstruction with bone transport distraction and implants after partial maxillectomy. J Oral Maxillofac Surg.

[7] Warburton G, Shihabi A, Ord RA (2015). Keratocystic Odontogenic Tumor (KCOT/OKC)-Clinical Guidelines for Resection. J Maxillofac Oral Surg.

[8] Fidele NB, Bing L, Sun Y, Wu T, Zheng Y, Zhao Y (2019). Management of mandibular odontogenic keratocyst through radical resection: report of 35 cases. Oncol Lett.

[9] Al-Moraissi EA, Dahan AA, Alwadeai MS, Oginni FO, Al-Jamali JM, Alkhutari AS (2017). What surgical treatment has the lowest recurrence rate following the management of keratocystic odontogenic tumor?: a large systematic review and meta-analysis. J Craniomaxillofac Surg.

[10] Kolokythas A, Fernandes RP, Pazoki A, Ord RA (2007). Odontogenic keratocyst: to decompress or not to decompress? A comparative study of decompression and enucleation versus resection/peripheral ostectomy. J Oral Maxillofac Surg.

[11] Al-Nawas B, Schiegnitz E (2014). Augmentation procedures using bone substitute materials or autogenous bone - a systematic review and meta-analysis. Eur J Oral Implantol.

[12] Verlinden CRA, van de Vijfeijken SECM, Tuinzing DB, Becking AG, Swennen GRJ (2015). Complications of mandibular distraction osteogenesis for acquired deformities: a systematic review of the literature. Int J Oral Maxillofac Surg.

[13] Schlund M, Touzet-Roumazeille S, Nicot R, Ferri J (2020). Temporomandibular joint ankylosis following mandibular distraction osteogenesis: a dreadful complication. J Craniofac Surg.

[14] Wang B, Zhai J, Zheng Y, Tong H, Lü Y, Chen Z (2021). Temporomandibular joint dislocation in patients with cleft lip and palate after maxillary distraction osteogenesis: three case reports. Medicine (Baltimore)..

[15] Uckan S, Veziroglu F, Arman A (2006). Unexpected breakage of mandibular midline distraction device: case report. Oral Surg Oral Med Oral Pathol Oral Radiol Endod.

[16] Dasukil S, Verma S, Boyina KK, Jena AK (2021). Unpredicted bilateral device breakage during active phase of mandibular distraction: a case report and literature review. J Stomatol Oral Maxillofac Surg.

[17] Lee JA, Park DH, Yoon SH, Chung J (2008). Distractor breakage in cranial distraction osteogenesis for children with craniosynostosis. Pediatr Neurosurg.

[18] Aikawa T, Iida S, Isomura ET, Namikawa M, Matsuoka Y, Yamada C (2008). Breakage of internal maxillary distractor: considerable complication of maxillary distraction osteogenesis. Oral Surg Oral Med Oral Pathol Oral Radiol Endod.

